# Association between migraine and venous thromboembolism: a Mendelian randomization and genetic correlation study

**DOI:** 10.3389/fgene.2024.1272599

**Published:** 2024-05-01

**Authors:** Xu-Peng Wu, Peng-Peng Niu, Hong Liu

**Affiliations:** ^1^ Department of Neurology, Heping Hospital Affiliated to Changzhi Medical College, Changzhi, China; ^2^ Department of Neurology, The First Affiliated Hospital of Zhengzhou University, Zhengzhou, China

**Keywords:** causal relationship, genetic correlation, migraine, Mendelian randomization, venous thromboembolism

## Abstract

**Objective:**

Previous observational studies have reported an increased risk of venous thromboembolism (VTE) among individuals with migraine. This study aimed to investigate the causal effect of migraine on the development of VTE, as well as explore the genetic correlation between them.

**Methods:**

We conducted a two-sample Mendelian randomization (MR) study using publicly available summary statistics from large-scale genome-wide association studies for migraine and VTE. Linkage disequilibrium score regression analysis was performed to estimate the genetic correlation between migraine and VTE.

**Results:**

There were several shared risk variants (*p*-value < 5 × 10^−8^) between migraine and VTE. Linkage disequilibrium score regression analysis found a significant positive genetic correlation between migraine and VTE. The genetic correlations based on two migraine datasets were 0.208 (se = 0.031, *p*-value = 2.91 × 10^−11^) and 0.264 (se = 0.040, *p*-value = 4.82 × 10^−11^), respectively. Although main MR analysis showed that migraine was associated with an increased risk of VTE (odds ratio = 1.069, 95% confidence interval = 1.022–1.118, *p*-value = 0.004), the association attenuated to non-significance when using several other MR methods and using another set of genetic instruments. In addition, evidence of heterogeneity was found. Reverse MR analysis showed VTE was associated with increased risk of migraine with aura (odds ratio = 1.137, 95% confidence interval = 1.062–1.218, *p*-value = 2.47 × 10^−4^) with no evidence of pleiotropy and heterogeneity.

**Conclusion:**

We showed suggestive evidence indicating an association between migraine and increased risk of VTE. Additionally, we found robust evidence suggesting that VTE is associated with an increased risk of migraine. The positive genetic correlation indicates that migraine and VTE has shared genetic basis. Further investigations will be necessary to address potential sex-specific effects in the analysis.

## Introduction

Migraine is a chronic neurological disorder characterized by recurring episodes of moderate to severe headache, often accompanied by other symptoms such as nausea, vomiting, and sensitivity to light and sound (Puledda et al., 2023). The headache typically presents as a pulsating or throbbing pain, usually on one side of the head. These attacks can last for hours or even days, causing substantial impairment in daily activities and quality of life for those affected.

Migraine affects people of all ages, sexes, and ethnic backgrounds. According to recent studies, it is estimated that approximately 1 in 10 individuals worldwide experience migraines (Woldeamanuel and Cowan, 2017). Women are more commonly affected than men, with hormonal fluctuations playing a significant role in triggering migraine attacks (Khandelwal et al., 2022).

In addition to the direct impact on individuals and society, migraine has been associated with an increased risk of developing other comorbid conditions. These can include cardiovascular diseases (Kalkman et al., 2023), depression, epilepsy, and anxiety disorders (Pelzer et al., 2023).

Venous thromboembolism (VTE), primarily comprising deep vein thrombosis and its associated complication, pulmonary embolism, has the potential to cause severe and life-threatening consequences (Lutsey and Zakai, 2023). Several observational studies have reported an increased risk of VTE among individuals with migraine (Bushnell et al., 2009; Wabnitz and Bushnell, 2015; Khandelwal et al., 2022). A large population based cohort study conducted by Adelborg et al. (2018) involving over 50,000 migraine participants found that migraine patients had a significantly higher risk of VTE compared to controls. The increased risk of VTE is similar between individuals with migraine with aura and migraine without aura. However, some studies have failed to find any association between migraine and VTE, while others have suggested that the association may be limited to patients who experience migraine with aura, rather than those without aura (Peng et al., 2016; Folsom et al., 2019).

Due to methodological limitations, traditional observational studies are unable to adequately establish a causal relationship. By leveraging genetic variants as instrumental variables, new methodology of Mendelian randomization (MR) approach can mitigate the causal and confounding biases encountered in traditional observational studies, thereby providing a more robust basis for establishing causal relationships (Hemani et al., 2018; de Leeuw et al., 2022).

In this study, we aimed to assess the causal effect of migraine on VTE using two-sample MR approach. To gain insight into the association, we employed linkage disequilibrium score regression (LDSC) analysis to assess the genetic correlation between migraine and VTE. LDSC approach allows us to examine the shared underlying genetic factors between these two conditions and provide insights into their potential biological connections.

## Materials and methods

This study utilized publicly accessible summary statistics data, with no identifiable information on participants. Consequently, ethical approval and informed consent were not required for this research. Guidelines for reporting MR studies were followed (Skrivankova et al., 2021). Table 1 shows the characteristics of included genome-wide association studies (GWASs).

**TABLE 1:** Characteristics of genome-wide association studies included.

Trait	Study	Data source	Sample size (cases/controls)	Ancestry	Exposure^a^	Outcome^a^	Trait definition
Any migraine	Hautakangas et al. (2022) ^b^	21 studies from Gormley et al. (2016)	29,209/172,931	European	Yes	No	Self-reported and ICHD-II
		23andMe	53,109/230,879	European	Yes	No	Self-reported
		UK biobank	10,881/330,170	European	Yes	No	Self-reported
		GenerISK	1,084/4,857	European	Yes	No	Self-reported
		HUNT	7,801/32,423	European	Yes	No	Self-reported and modified ICHD-II
Any migraine	Choquet et al. (2021)	UK biobank	17,532/465,435	European (94.19%)	Yes	Yes	Self-reported
		GERA cohort	11,320/60,282	European (81.53%)	Yes	Yes	A algorithm and ICD-8, ICD-9 and ICD-10
		21 studies from Gormley et al. (2016) ^b^	29,209/172,931	European	Yes	No	Self-reported and ICHD-II
Any migraine	FinnGen	FinnGen	18,477/287,837	European	No	Yes	ICD-8, ICD-9 and ICD-10
Migraine with aura	FinnGen	FinnGen	7,917/287,837	European	No	Yes	ICD-8, ICD-9 and ICD-10
Migraine with aura	Hautakangas et al. (2022) ^b^	12 studies from Gormley et al. (2016)	6,332/144,883	European	Yes	No	Self-reported and ICHD-II
		UK biobank	1,333/320,139	European	Yes	No	Self-reported
		deCODE	2,297/209,338	European	Yes	No	ICD10 G43 or ICPC-2 N89
		DBDS	3,938/28,045	European	Yes	No	Self-reported and ICHD-III
		LUMINA	724/1,447	European	Yes	No	ICHD-III
Migraine without aura	FinnGen	FinnGen	6,730/287,837	European	No	Yes	ICD-8, ICD-9 and ICD-10
Migraine without aura	Hautakangas et al. (2022) ^b^	11 studies from Gormley et al. (2016)	8,348/139,622	European	Yes	No	Self-reported and ICHD-II
		UK biobank	187/320,139	European	Yes	No	Self-reported
		deCODE	1,648/193,050	European	Yes	No	ICD-10 G43 or ICPC-2 N89
		DBDS	3,756/28,045	European	Yes	No	Self-reported and ICHD-III
		LUMINA	1,116/1,445	European	Yes	No	ICHD-III
Venous thromboembolism	Ghouse et al. (2023)	UK Biobank	23,723/412,717	European	Yes	Yes	Self-reported and ICD-10
		deCODE	11,700/311,745	European	Yes	Yes	ICD-9, ICD-10 and Nordic Surgical Procedure Codes
		Intermountain	6,981/51,252	European	Yes	Yes	ICD-10
		CHB-CVDS/DBDS	18,569/213,503	European	Yes	Yes	ICD-10
		FinnGen	11,288/249,117	European	Yes	Yes	ICD-9 and ICD-10
		MVP	8,929/181,337	European	Yes	Yes	ICD-9 and ICD-10

^a^ Yes indicates that the data was used as exposure/outcome data in the present Mendelian randomization study.

^b^ Full summary-data for these studies was not publicly accessible.

**Abbreviations**: CHB-CVDS, copenhagen hospital biobank cardiovascular disease cohort; DBDS, danish blood donor study; GERA, genetic epidemiology research in adult health and aging; HUNT, an acronym for the Norwegian name: Helseundersøkelsen i Nord-Trøndelag; ICHD, international classification of headache disorders; ICD, international classification of disease; LUMINA, leiden university migraine neuro analysis; MVP, million veterans programs.

### Assumptions of Mendelian randomization study

The MR approach utilizes instrumental variables, which are genetic variants randomly allocated at conception, to estimate the causal effect of an exposure on an outcome. Genetic variants act as natural experiments because they are randomly assigned during meiosis when gametes are formed. This random allocation mimics the process of randomization seen in controlled experiments and reduces the risk of confounding factors biasing the results.

**FIGURE 1:**
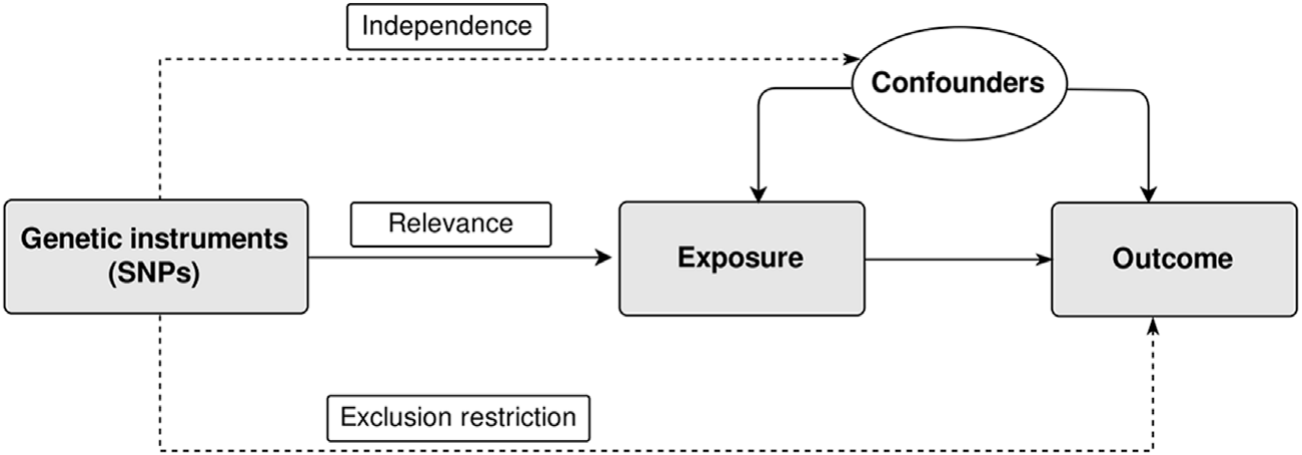
Assumptions of two-sample Mendelian randomization study. There are three core assumptions for two-sample Mendelian randomization study. Relevance: The genetic instrument is associated with the exposure; Independence: The genetic instrument is not associated with any confounders; Exclusion restriction: the genetic instrument does not affect outcome via pathways other than the exposure. SNP, single nucleotide polymorphism.

The MR approach relies on three key assumptions that are crucial for valid causal inference (Figure 1). (de Leeuw et al., 2022) These assumptions help to ensure that the genetic variants used as instrumental variables truly act as proxies for the exposure of interest and minimize biases in estimating causal effects. The three core assumptions of MR are:

1) Relevance: This assumption states that the genetic instruments used in MR are strongly associated with the exposure variable.
2) Independence: The independence assumption requires that the genetic instruments used in MR are independent of confounding factors.
3) Exclusion restriction: This assumption assumes that the genetic instruments used in MR only influence the outcome variable through their effect on the exposure of interest.

### Migraine dataset

Hautakangas et al. (2022) conducted a GWAS meta-analysis involving 102,084 migraine cases and 771,257 controls of European ancestry. There were five study cohorts in this study. All models in the individual study cohorts were adjusted for sex and, at minimum, the four leading principal components of the genetic population structure. Age adjustment was performed when the data was available. The GWAS meta-analysis was performed using an inverse-variance-weighted fixed-effect model by using GWAMA.

A total of 8,117 single nucleotide polymorphisms (SNPs) associated with migraine at genome-wide level (*p*-value < 5 × 10^−8^) were identified in this study. As a result, 123 risk loci were identified, and each locus may contain more than one risk SNPs. All SNPs in these 123 risk loci were independent from each other at a linkage disequilibrium threshold of *r* ^2^ < 0.1. The physical distances between each pair of loci were greater than 250 kb apart. The 123 SNPs with the lowest *p*-value in each locus were chosen as lead variants. We chose the 123 SNPs as candidate genetic instruments. We further performed a linkage disequilibrium clumping procedure using a wide, 10-Mb window and a stringent linkage disequilibrium threshold (*r* ^2^ = 0.01) to identify independently significant SNPs (Niu et al., 2022). The reference panel used was the European 1,000 Genome Project v3. Next, two SNPs located in the major histocompatibility complex region were further excluded. In total, 113 SNPs were chosen as genetic instruments (Supplementary Table S1).

The authors additionally reported the associations between the 123 SNPs and specific migraine subtypes, namely, migraine with aura (14,624 cases and 703,852 controls) and migraine without aura (15,055 cases and 682,301 controls). Since there were a limited number of loci associated with migraine subtypes, we relaxed the significance threshold to *p*-value < 1.0 × 10^−5^. This strategy is commonly employed in previous MR studies (Lee et al., 2022; Cao et al., 2023; Chen et al., 2024). In addition, because this study only reported SNP effects of the 123 SNPs on migraine subtypes, we further included SNPs associated with each migraine subtype at a *p*-value threshold of < 1 × 10^−5^ from another meta-analysis of GWASs to expanded the number of genetic instruments for migraine subtypes. Following the clumping procedure, we identified 47 SNPs associated with migraine with aura and 46 SNPs associated with migraine without aura (*p*-value < 1.0 × 10^−5^), which were subsequently included as genetic instruments in our MR analysis (Supplementary Table S2).

In addition, reverse MR analysis was performed to assess the causal effects of VTE on migraine. We utilized migraine datasets from Choquet et al. (2021) and the FinnGen consortium as the outcome data for our analysis.

Choquet et al. (2021) conducted a multiethnic GWAS meta-analysis of migraine, combining results from the Genetic Epidemiology Research in Adult Health and Aging (GERA) cohort (11,320 cases and 60,282) and the UK Biobank cohort (17,532 cases and 465,435 controls). Most of the individuals included in the study were of European ancestry. A logistic regression model was employed for each GWAS, adjusting for age, sex, and leading ancestry principal components. The combined multiethnic (GERA + UK Biobank) meta-analysis GWAS summary statistics was used as one of the outcome data for migraine.

Choquet et al. (2021) conducted a European ancestry meta-analysis of 85,726 migraine cases and 803,292 controls. Besides individuals from the GERA cohort and the UK Biobank cohort, 59,674 migraine cases and 316,078 controls from the study of Gormley et al. (2016) were included. The meta-analysis identified 73 independent risk loci. Following the clumping procedure, we used the 69 remained SNPs as a second set of genetic instruments to perform a sensitivity analysis for the main MR analysis (Supplementary Table S1).

Migraine summary data from the FinnGen study was used as one of the outcome data for migraine. The latest release (Release 9) of the FinnGen study included 18,477 migraine cases and 287,837 controls. In addition, the summary data for migraine subtypes were also available. There were 7,917 cases of migraine with aura and 6,730 cases of migraine without aura. Sex, age, 10 ancestry principal components, and genotyping batch were adjusted in the GWASs in the FinnGen study (Kurki et al., 2023).

### Venous thromboembolism dataset

Ghouse et al. (2023) performed a GWAS meta-analysis of VTE incorporating 81,190 cases and 1,419,671 controls of European descent sampled from six cohorts. In these cohorts, cases of VTE were identified based on hospital or register records using the International Classification of Diseases (ICD)-9 or ICD-10 coding system. Additionally, in the UK Biobank cohort, individuals who self-reported a diagnosis of VTE were also included as cases. The VTE cases mainly comprise pulmonary embolism (ICD-10 code: I26), obstetric embolism (ICD-10 code: O882), phlebitis and thrombophlebitis (ICD-10 code: I80), as well as other venous embolism or thrombosis (ICD-10 code: I82). At least age, sex and ancestry principal components were included as covariates. The GWAS meta-analysis was performed using the fixed-effect inverse-variance-weighted method by using METAL. For SNPs that were not found in the VTE GWAS, a proxy SNP was used if it had a linkage disequilibrium measure, specifically an *r* ^2^ value, greater than 0.8.

In this study, risk loci were defined as a region of ±1,000 kb around each lead SNP. All SNPs within each risk locus were independent from each other (*r* ^2^ = 0.01, 5000 kb). At last, the authors identified 93 risk loci. We chose the 93 lead SNPs as candidate genetic instruments. We excluded two SNPs located in the major histocompatibility complex region. We further performed a linkage disequilibrium clumping procedure using common used parameters (*r* ^2^ = 0.01, 10,000 kb). At last, a total of 85 SNPs were chosen as genetic instruments for the reverse MR study (Supplementary Table S3).

### Instrument strength and statistical power of MR analysis

The F statistic was computed for each SNP using the following formula: F = beta (Woldeamanuel and Cowan, 2017)/se (Woldeamanuel and Cowan, 2017), where beta represents the estimate of the effect of the SNP on the exposure, and se represents the standard error of the estimate. If SNPs with an F statistic < 10 are included, it may introduce a weak instrumental bias. In the present analysis, all the genetic instruments included had F statistics > 10.

The formula used to calculate the proportion of variance explained by each SNP is as follows: *R* ^2^ = 2 ×beta (Woldeamanuel and Cowan, 2017) × MAF × (1–MAF) (Park et al., 2010), MAF indicates the minor allele frequency. For primary analysis of any migraine on VTE, the total variance explained of SNPs used in the MR analysis was 8.43%. There was an 80% power to detect an odds ratio (OR) of 1.035 with a type-Ⅰ error rate of 5% (Brion et al., 2013). For primary analysis of migraine with aura on VTE, there was an 80% power to detect an OR of 1.020. For primary analysis of migraine without aura on VTE, there was an 80% power to detect an OR of 1.021. In addition, the detectable ORs of VTE on any migraine by Choquet et al. (2021), any migraine by FinnGen, migraine with aura and migraine without aura under an 80% power were 1.043, 1.061, 1.091 and 1.098, respectively (Supplementary Table S4).

### Mendelian randomization analysis

The primary method employed for MR analysis was the random-effects inverse-variance weighted approach. It assumes that all instrumental variables are valid and unbiased, with no measurement error or heterogeneity. Even if there is some degree of pleiotropy among the instrumental variables, as long as there is no evident imbalance, the IVW method can provide unbiased causal estimates. However, it may still be subject to other potential biases. In addition, it's important to note that identifying balanced pleiotropy is challenging, and it is difficult to establish whether pleiotropy is truly balanced in practice. MR-Egger method allows for the detection and correction of potential bias due to pleiotropic effects, providing more robust estimates of causal effects. The weighted median method provides a consistent causal estimate as long as more than 50% of the instrumental variables are valid. The weighted mode method is used to estimate causal effects when there is uncertainty about the validity of IVs. This method takes into account the possibility of a mixture of valid and invalid instruments. We used MR-PRESSO to identify instrumental variables that deviate significantly from the null hypothesis of no pleiotropy (Verbanck et al., 2018). We next utilized the generalized summary-data-based MR (GSMR) method to evaluate the potential causal associations (Zhu et al., 2018). GSMR offers greater power compared to the above MR methods due to its ability to account for sampling variance in both SNP-exposure association and SNP-outcome association. Unlike other approaches, GSMR does not assume that SNP-exposure association is estimated without error, thereby providing more robust results (Zhu et al., 2018). The HEIDI-outlier analysis is a method for identifying and removing genetic instruments that exhibit potential pleiotropic effects. SNPs that pass the HEIDI-outlier analysis were subsequently included in the final GSMR analysis. To address the issue of overlapping samples and explicitly consider study heterogeneity, we employed Bayesian MR analysis (Zhao et al., 2020; Zou et al., 2022).

To assess potential heterogeneity between individual SNPs, the Cochrane’s Q test was utilized for the IVW method, while Rücker’s Q test was employed for the MR-Egger method (Bowden et al., 2018). The MR-Egger regression intercept was employed as a measure to assess the presence of horizontal pleiotropy.

We conducted MR Steiger test to examine the directionality between the exposure and outcome, assessing the validity of the assumption that the exposure causes the outcome. Additionally, we utilized a Steiger filtering procedure to eliminate potential reverse causal instruments (Hemani et al., 2017). For the sensitivity analysis, only the SNPs with a low probability of reverse causality (Steiger *p*-value < 0.05) were included. Isolated populations, such as the Finnish population, possess distinct genetic backgrounds that can lead to a higher occurrence of rare and low-frequency variants with deleterious effects compared to other European populations (Kääriäinen et al., 2017). We conducted a sensitivity analysis exclusively utilizing data from FinnGen. We employed the Bayesian MR method to mitigate the potential confounding effects of sample overlap.

MR analysis was conducted using the R packages TwoSampleMR, gwasglue, gwasvcf, MR-PERESSO, GSMR and BWMR (Zhao et al., 2020) in R (version 3.6.1). We considered a Bonferroni-corrected significance level of *p*-value < 0.008 (0.05/6; accounting for 3 migraine exposures and 3 migraine outcomes) as indicating statistical significance. *p*-value falling between 0.008 and 0.05 was regarded as suggestive associations.

### Linkage disequilibrium score regression

To assess the genetic correlation between migraine and VTE, the LD score software (LDSC v1.0.1) was utilized (Bulik-Sullivan et al., 2015). LDSC provides a powerful framework for estimating genetic correlations using summary statistics from GWAS. Pre-calculated LD scores that did not include any variants located within the major histocompatibility complex region were used. The pre-calculated LD scores were based on 1,000 Genomes European data. SNPs with Chi-square values greater than 80 were excluded from the analysis because the regression can be unduly influenced by outliers (Zheng et al., 2017).

The regression analysis is performed by regressing the cross-product of two z-scores obtained from two GWASs on the LD scores. The estimated genetic covariance between two traits is obtained by using the slope derived from this regression.

## Results

### Mendelian randomization analysis of migraine on VTE

Among the 113 SNPs associated with any migraine, one of them was not found in the VTE dataset. Six SNPs associated with VTE at genome-wide level (*p*-value < 5 × 10^−8^) were excluded from the MR analysis (Supplementary Table S1). The effect alleles of five out of the six SNPs present the same direction of effects on both migraine and VTE. Next, five palindromic SNPs with intermediate allele frequency were excluded. A total of 101 SNPs including two proxies (*r* ^2^ > 0.8) (Supplementary Table S3) were used in the final MR analysis.

**FIGURE 2:**
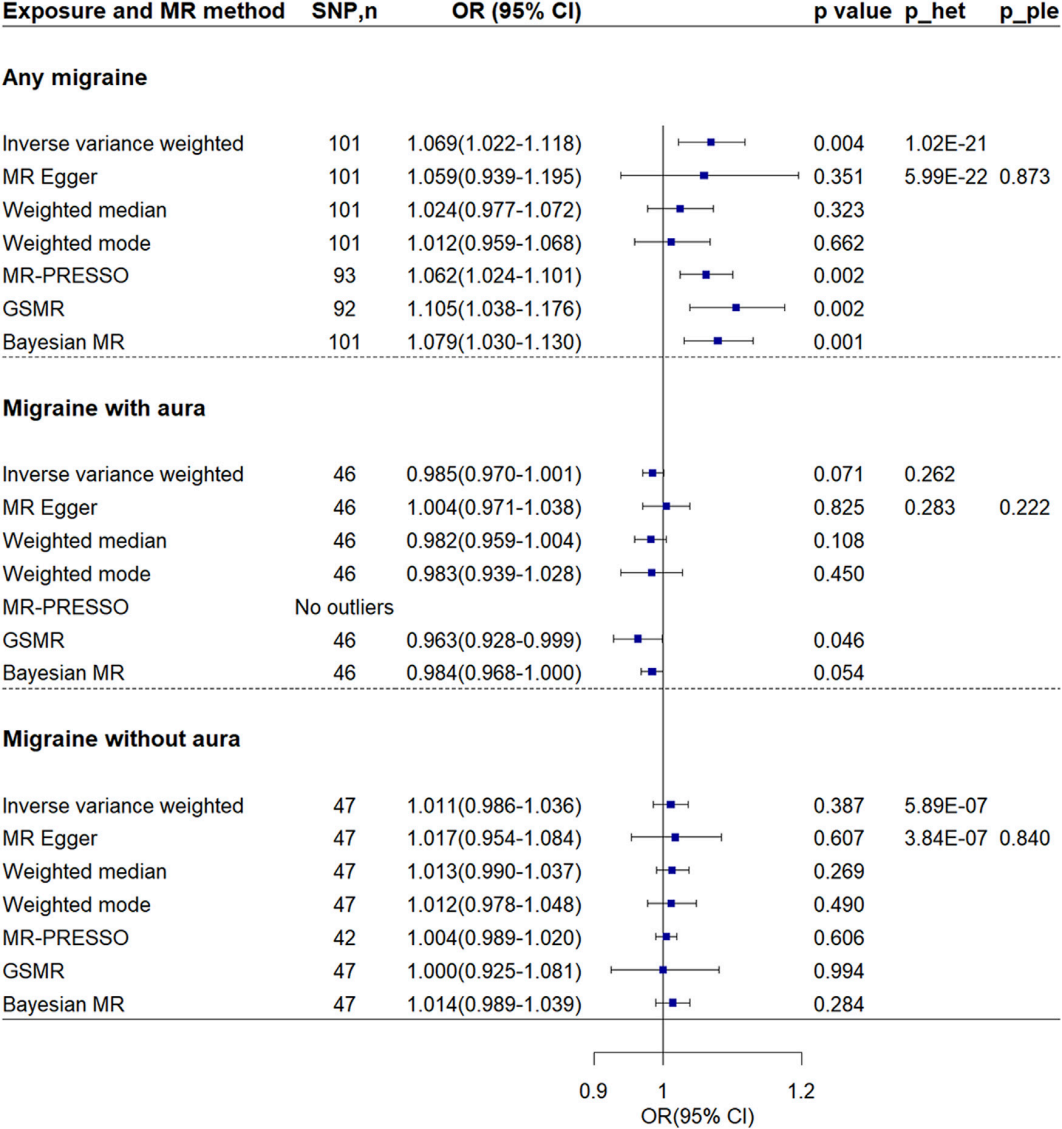
Mendelian randomization analyses of migraine on venous thromboembolism. P_ple indicates *p*-value of MR-Egger regression intercept; p_het indicates *p*-value of heterogeneity test. Abbreviations: CI, confidence interval; MR, Mendelian randomization; GSMR, generalized summary-data-based MR; MR-PRESSO, MR-Pleiotropy Residual Sum and Outlier; OR, odds ratio; SNP, single nucleotide polymorphism.

**FIGURE 3:**
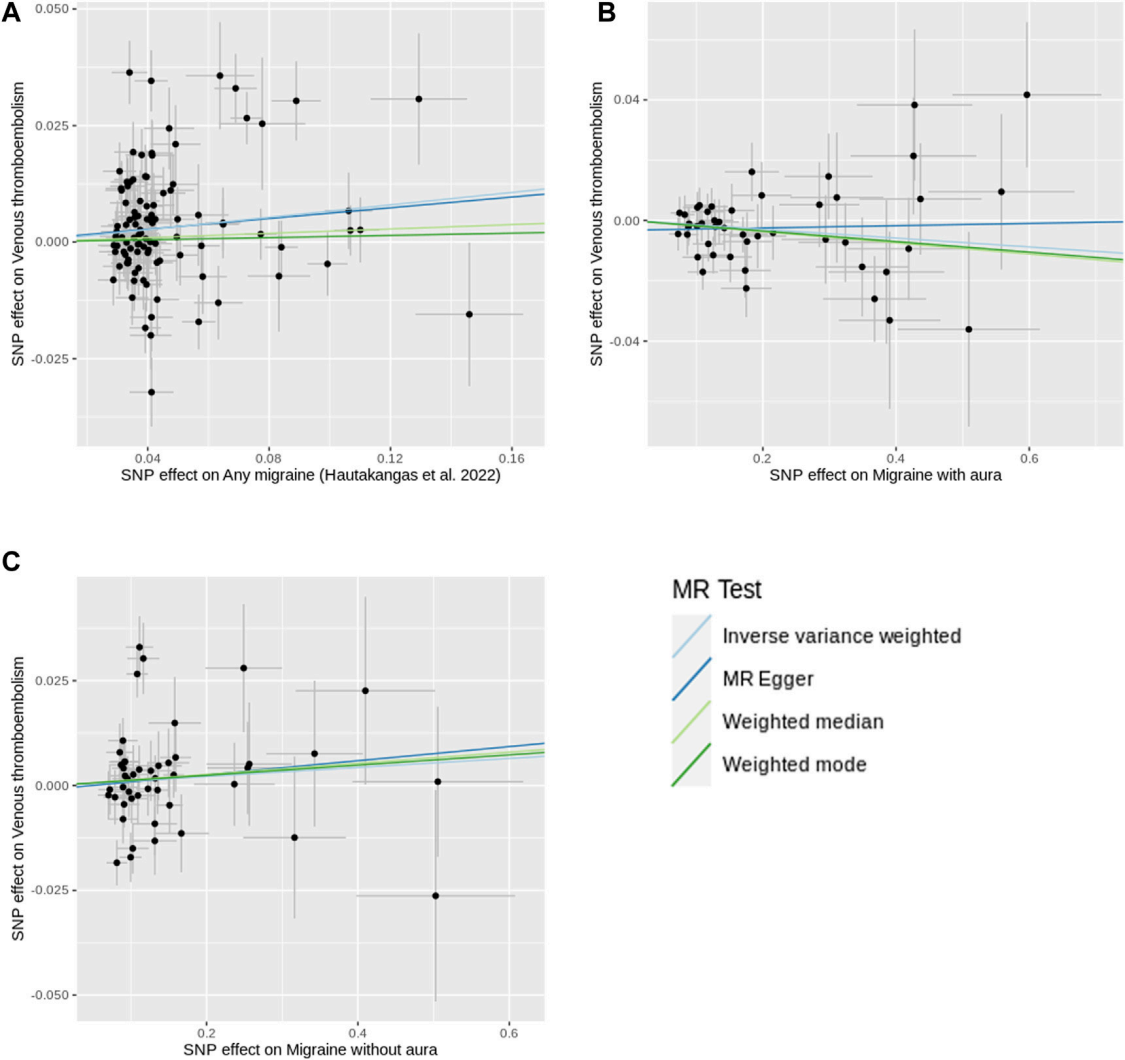
Scatter plots of SNP effects on migraine and venous thromboembolism. **(A)** shows the plot using any migraine data. **(B)** shows the plot using migraine with aura data. **(C)** shows the plot using migraine without aura data. X-axes represent SNP effects on migraine. Y-axes represent SNP effects on venous thromboembolism. Abbreviations: MR, Mendelian randomization; SNP, single nucleotide polymorphism.

Inverse-variance weighted method showed migraine was associated with increased risk of VTE [OR = 1.069, 95% confidence interval (CI) = 1.022–1.118, *p*-value = 0.004] (Figures 2, 3). The association was confirmed through both GSMR and Bayesian MR analyses. Leave-one-out analysis showed the result was not driven by any singly SNP. MR-PRESSO method showed the association remained after excluding 8 outliers. However, the association attenuated to non-significant when using other MR methods. Although MR-Egger regression showed no evidence of unbalanced pleiotropy (*p*-value = 0.873), significant heterogeneity was found (*p*-value = 1.02 × 10^−21^ and 5.99 × 10^−22^). The significant heterogeneity remained when the 8 outliers were excluded (*p*-value = 1.21 × 10^−5^ and 1.11 × 10^−5^). And estimates from the MR-Egger (OR = 1.028, 95% CI = 0.935–1.130, p-vlaue = 0.573), weighted median (OR = 1.024, 95% CI = 0.977–1.072, *p*-value = 0.573), and weighted mode (OR = 1.012, 95% CI = 0.958–1.069, *p*-value = 0.667) methods kept essentially unchanged when the 8 outliers were excluded.

MR Steiger test confirmed the directionality between the any migraine and VTE (Steiger *p*-value = 9.40 × 10^−267^). In the sensitivity analysis using the inverse-variance weighted method and considering the 95 SNPs with a low probability of reverse causality (Steiger *p*-value < 0.05), the association between any migraine and VTE attenuated but remained suggestive significant (OR = 1.055, 95% CI = 1.014–1.098, *p*-value = 0.008) (Supplementary Table S5). Sensitivity analysis by using another set of SNPs showed the association was not significant (OR = 1.048, 95% CI = 0.993–1.106, *p*-value = 0.090) (Supplementary Figures S1, S2). Except for the Bayesian MR method, all other MR methods showed no association. Although no evidence of unbalanced pleiotropy was found, evidence of heterogeneity was found.

When exclusively analyzing data from FinnGen and employing genetic instruments with a *p*-value threshold of < 1.00 × 10^−5^, our Bayesian MR analysis revealed a suggestive association between migraine and an increased risk of VTE (OR = 1.068, 95% CI = 1.010–1.129, *p*-value = 0.021) (Supplementary Figure S3).

Using genetic instruments at a p-level of < 1 × 10^−5^, the MR analyses indicated that both migraine with aura (OR = 0.985, 95% CI = 0.970–1.001, *p*-value = 0.071) and migraine without aura (OR = 1.011, 95% CI = 0.986–1.036, *p*-value = 0.387) were not associated with the risk of VTE (Figures 2, 3). No evidence of heterogeneity and pleiotropy was found for migraine with aura. No SNP shows a probability of reverse causality, as all Steiger *p*-values were less than 0.05. Although GSMR showed migraine with aura was suggestively associated with decreased VTE risk (OR = 0.963, 95% CI = 0.928–0.999, *p*-value = 0.046), the association was dismissed when conducting sensitivity analyses using genetic instruments at the genome-wide level (*p*-value < 5 × 10^−8^) (Supplementary Figures S1, S2). Upon exclusive analysis of data from FinnGen, the Bayesian MR method indicated a suggestive increase in the risk of VTE for individuals with migraine without aura (OR = 1.050, 95% CI = 1.010–1.092, *p*-value = 0.014) (Supplementary Figure S3). However, no association was observed between migraine with aura and VTE risk (OR = 1.029, 95% CI = 0.981–1.080, *p*-value = 0.240).

### Reverse Mendelian randomization analysis of VTE on migraine

**FIGURE 4:**
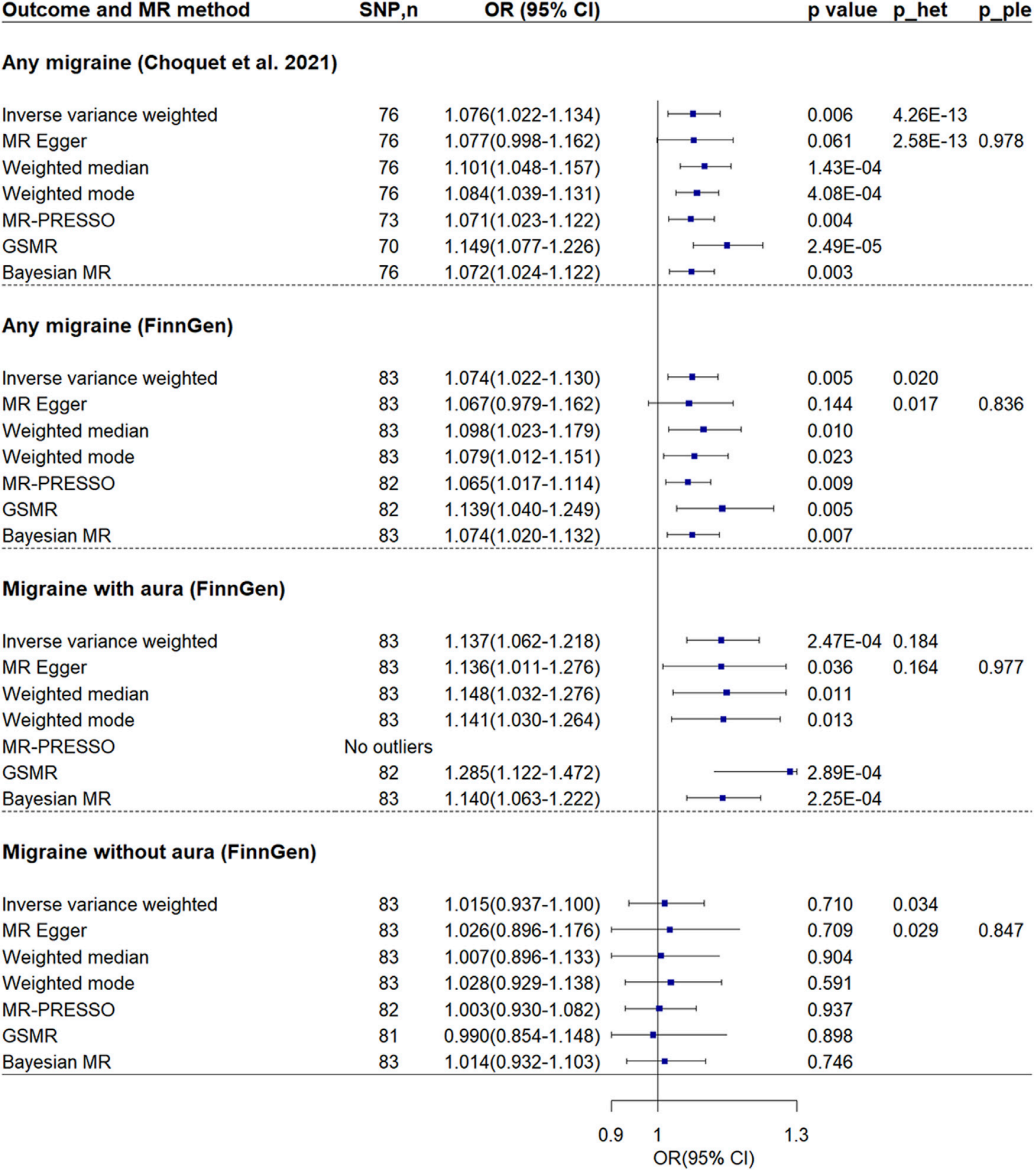
Mendelian randomization analyses of venous thromboembolism on migraine. P_ple indicates *p*-value of MR-Egger regression intercept; p_het indicates *p*-value of heterogeneity test. Abbreviations: CI, confidence interval; MR, Mendelian randomization; GSMR, generalized summary-data-based MR; MR-PRESSO, MR-Pleiotropy Residual Sum and Outlier; OR, odds ratio; SNP, single nucleotide polymorphism.

Reverse MR analysis showed VTE was associated with increased risk of migraine (Figure 4; Supplementary Figure S4). The ORs in two separate migraine datasets were 1.076 (95% CI = 1.022–1.134, *p*-value = 0.006) and 1.074 (95% CI = 1.022–1.130, *p*-value = 0.005) respectively. Note there was no sample overlap between the two migraine datasets. No evidence of unbalanced pleiotropy was found. Although significant heterogeneity was found, all other MR methods showed similar estimates.

Further MR analysis revealed that the effect was found to be significant only in individuals with migraine accompanied by aura (OR = 1.137, 95% CI = 1.062–1.218, *p*-value = 2.47 × 10^−4^), while it was not found to be significant in individuals without aura (OR = 1.015, 95% CI = 0.937–1.100, *p*-value = 0.710). In addition, no evidence of unbalanced pleiotropy and significant heterogeneity was found in analysis of migraine with aura.

MR Steiger test confirmed the directionality between the VTE and migraine. In the sensitivity analysis using the inverse-variance weighted method and considering the SNPs with a low probability of reverse causality (Steiger *p*-value < 0.05), the association between VTE and any migraine, particularly between VTE and migraine with aura, remained significant (Supplementary Table S5).

Upon exclusive analysis of data from FinnGen, the Bayesian MR method indicated a suggestive increase in the risk of any migraine for individuals with VTE (OR = 1.037, 95% CI = 1.000–1.074, *p*-value = 0.048) (Supplementary Figure S5). However, no association was observed between VTE and migraine with aura (OR = 1.041, 95% CI = 0.990–1.095, *p*-value = 0.119) or migraine without aura (OR = 1.010, 95% CI = 0.957–1.066, *p*-value = 0.709).

### Linkage disequilibrium score regression

There was evidence of a significant positive genetic correlation between migraine and VTE. The genetic correlation was 0.208 (se = 0.031, *p*-value = 2.91 × 10^−11^) and 0.264 (se = 0.040, *p*-value = 4.82 × 10^−11^) when using two separate migraine datasets with no sample overlap. The positive genetic correlations persist between VTE and both migraine with aura (genetic correlation = 0.267, se = 0.053, *p*-value = 4.30 × 10^−7^) as well as migraine without aura (genetic correlation = 0.262, se = 0.048, *p*-value = 5.37 × 10^−8^).

Upon conducting dedicated analysis using data exclusively from FinnGen, we found evidence of significant positive genetic correlations between VTE and migraine. Specifically, we observed a genetic correlation of 0.342 (se = 0.071, *p*-value = 1.32 × 10^−6^) for any migraine, a genetic correlation of 0.302 (se = 0.098, *p*-value = 0.002) for migraine with aura, and a genetic correlation of 0.344 (se = 0.076, *p*-value = 6.53 × 10^−6^) for migraine without aura.

## Discussion

In this study, our MR analysis showed that migraine was associated with an increased risk of VTE. However, the association attenuated to non-significance when using several other MR methods and using a different set of genetic instruments. Furthermore, the MR analyses indicated that both migraine with aura and migraine without aura were not associated with the risk of VTE. Interestingly, our reverse MR analysis revealed that VTE was associated with an increased risk of migraine. This association remained significant in individuals with migraine accompanied by aura but was not significant in individuals without aura. At last, LDSC analysis demonstrated a significant positive genetic correlation between migraine and VTE. Based on our analyses, it seems that the previously reported association between migraine and VTE is at least partly attributed to both shared underlying genetic factors and reverse causality.

Epidemiological studies have highlighted a potential link between migraine and vascular disorders, including coronary heart disease, stroke, as well as VTE (Bigal et al., 2009; Ng et al., 2022; Hvitfeldt Fuglsang et al., 2023). The associations appeared to be stronger in migraine with aura than in migraine without aura, in women than in men (Ng et al., 2022; Hvitfeldt Fuglsang et al., 2023). A systematic review and meta-analysis including 18 prospective cohort studies with 370,050 migraine patients and 1,387,539 controls showed migraine, especially migraine with aura, is associated with myocardial infarction and stroke (Ng et al., 2022). In addition, migraine with aura increases the risk of overall cardiovascular mortality. A nationwide, population-based cohort study was conducted involving 51,032 patients with migraine and 510,320 individuals from the general population (Adelborg et al., 2018). The individuals without a history of migraine were selected as study participants based on their age, sex, and calendar year. This research demonstrated a positive association between migraine and various cardiovascular conditions, including myocardial infarction (adjusted hazard ratio 1.49, 95% confidence interval 1.36–1.64), ischemic stroke (2.26, 2.11–2.41), hemorrhagic stroke (1.94, 1.68–2.23), venous thromboembolism (1.59, 1.45–1.74), as well as atrial fibrillation or atrial flutter (1.25, 1.16–1.36).

The underlying reasons for the association between migraine and vascular disorders may vary among different types of vascular diseases. Spreading depolarizations likely play a crucial role in the heightened risk of ischemic stroke observed in individuals with migraine, particularly those experiencing migraine with aura (Pelzer et al., 2023). Migraine with aura is associated with a higher prevalence of patent foramen ovale compared to migraine without aura (46.3%–88% vs. 16.2%–34.9%) (Liu et al., 2020). This increased prevalence of patent foramen ovale may potentially result in paradoxical embolism, subsequently increasing the risk of cerebral or coronary ischemic events. Patients with migraine have a higher occurrence of risk factors such as hypertension, diabetes, and hyperlipidemia, which are linked to various cardiovascular diseases (Bigal et al., 2009). Furthermore, there may be a connection between migraine and various vascular diseases through factors like endothelial dysfunction, hypercoagulability, platelet aggregation, and the use of non-steroidal anti-inflammatory drugs (Adelborg et al., 2018). At last, it has been suggested that the risk of VTE may be heightened due to immobilization during migraine attacks (Adelborg et al., 2018; Tasnim et al., 2023).

Although multiple observational studies have suggested a potential link between migraines and an increased risk of cardiovascular diseases, establishing a causal relationship has remained challenging due to confounding and reverse causality. Several studies have employed MR method to investigate the relationship between migraine and cardiovascular diseases. However, none of these MR studies were able to confirm an increased risk of migraine on cardiovascular diseases (Daghlas et al., 2020; Lee et al., 2022; Shu et al., 2022). The results even suggested a potentially protective causal effect of migraine on the risk of coronary artery disease and large-artery atherosclerosis stroke. Our MR study showed suggestive evidence indicating an association between migraine and increased risk of VTE. In addition, the observed associations between migraine and vascular disorders might be partly attributed to the shared risk factors. Previous LDSC analyses have revealed that migraine shares a genetic basis with cardiovascular risk factors, such as type 2 diabetes, lipid levels, and blood pressure (Guo et al., 2020; Siewert et al., 2020; Tanha et al., 2021a). However, there was no significant genetic correlation found between migraine and coronary artery disease or stroke (Siewert et al., 2020). Our LDSC analysis revealed a significant positive genetic correlation between migraine and VTE, supporting their potential biological connections. Furthermore, we identified several shared risk loci between migraine and VTE. These findings suggest that the observed associations between migraine and VTE may be partially attributed to the presence of genetic pleiotropy, where genetic variants associated with migraine may also influence the risk of VTE through shared biological pathways (Tanha et al., 2021b).

Interestingly, our reverse MR analysis revealed that VTE was associated with an increased risk of migraine. In addition, this effect was consistent across various migraine datasets. No evidence of heterogeneity and unbalanced pleiotropy was found. Given that VTE is associated with hypercoagulability and platelet aggregation, individuals who have a genetically increased risk of VTE may be more susceptible to paradoxical cerebral embolism through a potential right-to-left shunt. Therefore, in addition to the shared genetic basis, paradoxical cerebral embolism could potentially serve as a link between VTE and migraine. Platelet aggregate microembolization can potentially trigger focal transient ischemia, leading to the induction of cortical spreading depression. Cortical spreading depression is widely considered to be the electrophysiological mechanism behind aura in migraine (Finocchi and Del Sette, 2015).

Our study has several limitations. First, the use of self-reported cases from different datasets may introduce potential biases. Second, the inferences for migraine subtypes on VTE were limited due to the availability of a small number of genetic instruments meeting the significance threshold of *p*-value < 5 × 10^−8^. Third, the prevalence of migraine is significantly higher in women than in men. There may be sex differences in the association between migraine and VTE, but we do not have data segregated by sex to validate this. Fourth, several VTE types including pulmonary embolism, obstetric embolism, phlebitis and thrombophlebitis, along with other venous embolism or thrombosis, were incorporated into the VTE GWAS. The association between VTE and migraine might vary across different VTE subtypes. However, we have no data on VTE subtypes to carry out further analysis. Fifth, our research data primarily comes from individuals of European descent, and therefore, the results may not be directly applicable or generalizable to other racial or ethnic groups.

## Conclusion

Although our main analysis showed that migraine was associated with an increased risk of VTE, the association attenuated to non-significance when using several other MR methods and a different set of genetic instruments. This finding indicates association between migraine and an increased risk of VTE. Our reverse MR analysis revealed that VTE was associated with an increased risk of migraine. This association remained significant in individuals with migraine accompanied by aura but was not significant in individuals without aura. LDSC analysis demonstrated a significant positive genetic correlation between migraine and VTE. Based on our analyses, it seems that the association between migraine and VTE is at least partly attributed to biological pleiotropy (horizontal pleiotropy) and reverse causality. These findings provide additional support for the shared biological mechanisms between migraine and VTE. Further investigation including sex-specific GWAS data is highly anticipated.

## Data Availability

The original contributions presented in the study are included in the article/Supplementary Material, further inquiries can be directed to the corresponding authors.

## Abbreviations

CI, confidence interval; GWAS, genome wide association study; LDSC, linkage disequilibrium score regression; MR, Mendelian randomization; GSMR, generalized summary-data-based MR; MR-PRESSO, MR-Pleiotropy Residual Sum and Outlier; OR, odds ratio; SNP, single nucleotide polymorphism; VTE, venous thromboembolism.
