# Supplementary material for: Association between migraine and venous thromboembolism: a Mendelian randomization and genetic correlation study
Source: Front Genet. 2024 May 1;15:1272599. doi: 10.3389/fgene.2024.1272599 (PMC11097659; doi:10.3389/fgene.2024.1272599)
Supplement: Supplementary file 2 [file DataSheet1.docx]

**Supplementary Figures**

**Supplementary Figure 1** Sensitivity analyses of migraine on venous thromboembolism

**Supplementary Figure 2** Scatter plots of sensitivity analyses of migraine on venous thromboembolism

**Supplementary Figure 3** Scatter plots of migraine on venous thromboembolism by exclusively analyzing data from FinnGen

**Supplementary Figure 4** Scatter plots of SNP effects on venous thromboembolism and migraine

**Supplementary Figure 5** Scatter plots of venous thromboembolism on migraine by exclusively analyzing data from FinnGen


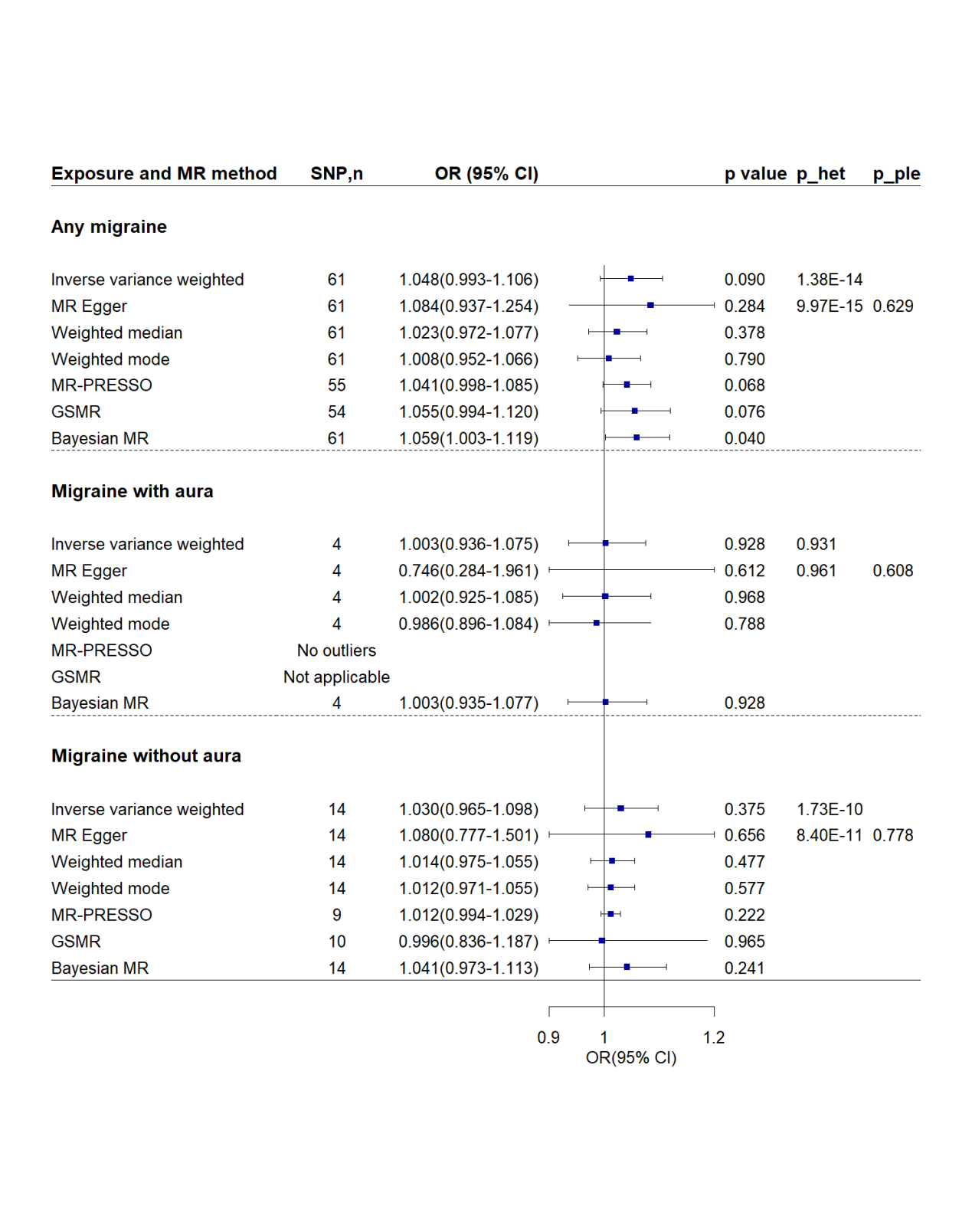


**Supplementary Figure 1 Sensitivity analyses of migraine on venous thromboembolism**

For any migraine, the significant SNPs (p-value < 5 × 10^-8^) identified by Choquet et al. which originated from 85,726 migraine cases and 803,292 controls were used to perform the sensitivity analysis.

For both migraine with aura and migraine without aura, sensitivity analyses were carried out by employing genetic instruments at the genome-wide level (p-value < 5 × 10^-8^), which differs from the primary analysis in which SNPs at a p-value of < 1 × 10^-5^ were utilized.

P_ple indicates p value of MR-Egger regression intercept; p_het indicates p value of heterogeneity test.

Abbreviations: CI, confidence interval; MR, Mendelian randomization; GSMR, generalized summary-data-based MR; MR-PRESSO, MR-Pleiotropy Residual Sum and Outlier; OR, odds ratio; SNP, single nucleotide polymorphism


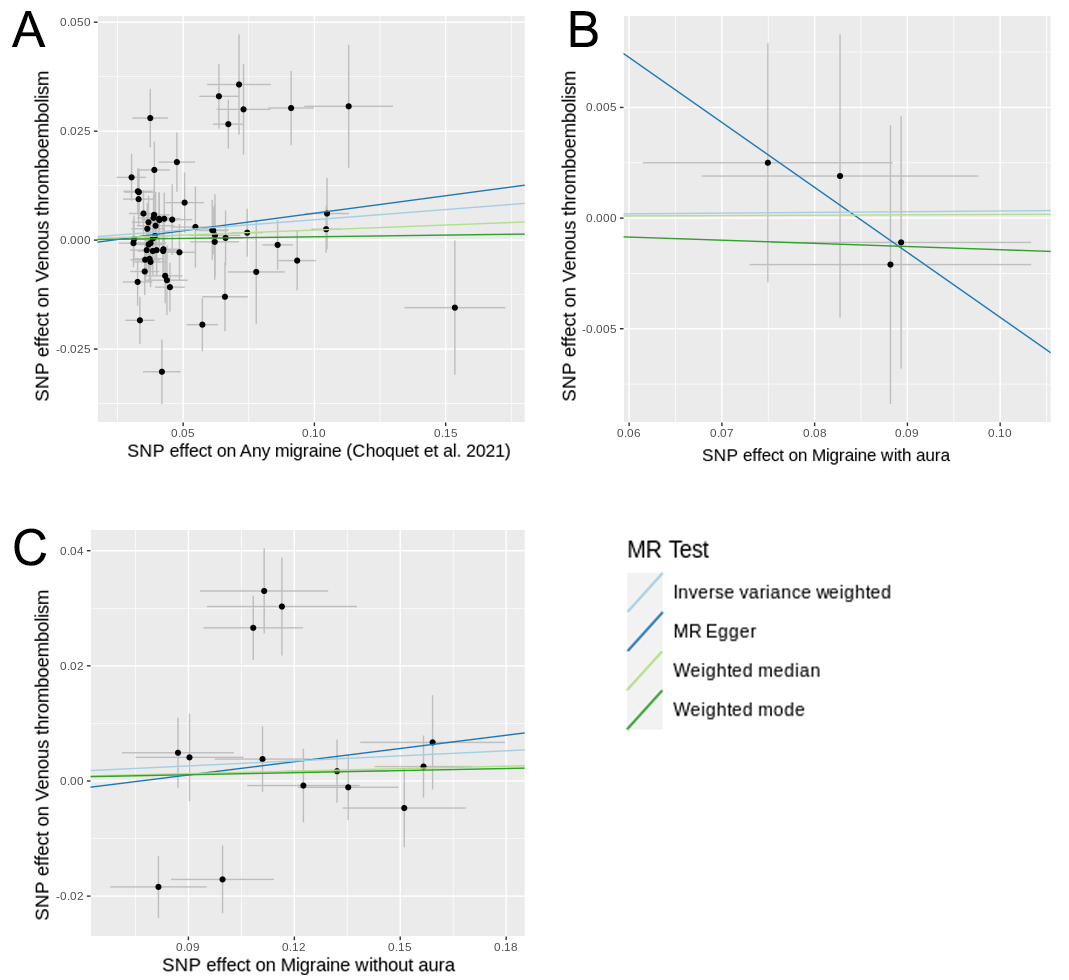


**Supplementary Figure 2 Scatter plots of sensitivity analyses of migraine on venous thromboembolism**

For any migraine, the significant SNPs (p-value < 5 × 10^-8^) identified by Choquet et al. which originated from 85,726 migraine cases and 803,292 controls were used to perform the sensitivity analysis.

For both migraine with aura and migraine without aura, sensitivity analyses were carried out by employing genetic instruments at the genome-wide level (p-value < 5 × 10^-8^), which differs from the primary analysis in which SNPs at a p-value of < 1 × 10^-5^ were utilized.

X axes represent SNP effects on migraine. Y axes represent SNP effects on venous thromboembolism.

Abbreviations: MR, Mendelian randomization; SNP, single nucleotide polymorphism

**
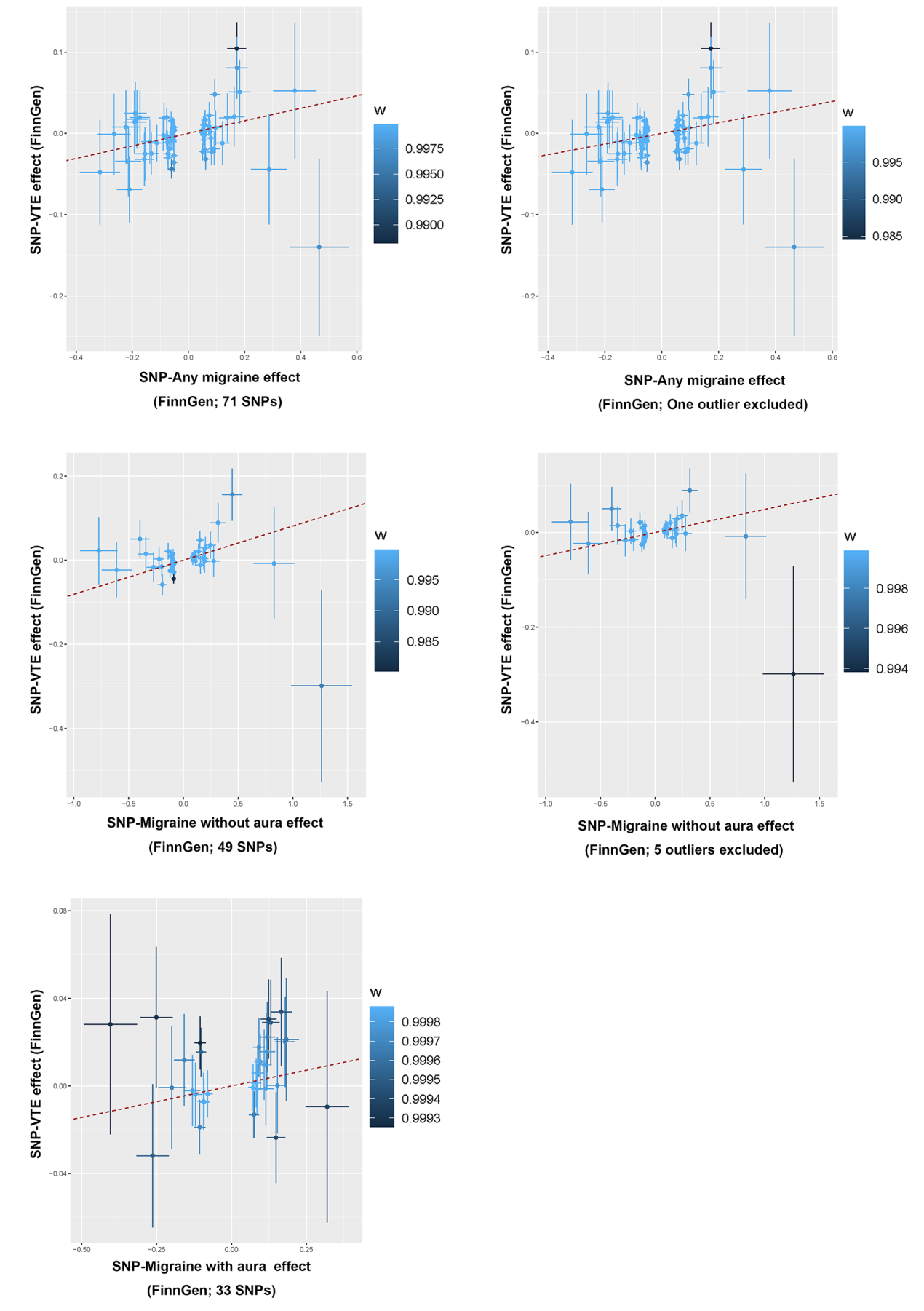
**

**Supplementary Figure 3 Scatter plots of migraine on venous thromboembolism by exclusively analyzing data from FinnGen.**

The Bayesian MR method was used.

Given that only three independent SNPs reached genome-wide significance for migraine (p-value < 5 × 10^-8^), we adjusted the significance threshold to p-value < 1.0 × 10^-5^ in order to capture additional variants. For any migraine, one outlier SNP was detected by MR-Pleiotropy Residual Sum and Outlier method. For migraine without aura, five SNPs without a low probability of reverse causality (Steiger p-value < 0.05) were detected.

For migraine with aura, no outlier was detected by MR-Pleiotropy Residual Sum and Outlier method. All SNPs have a low probability of reverse causality (Steiger p-value < 0.05).

Abbreviations: MR, Mendelian randomization; SNP, single nucleotide polymorphism; VTE, venous thromboembolism.


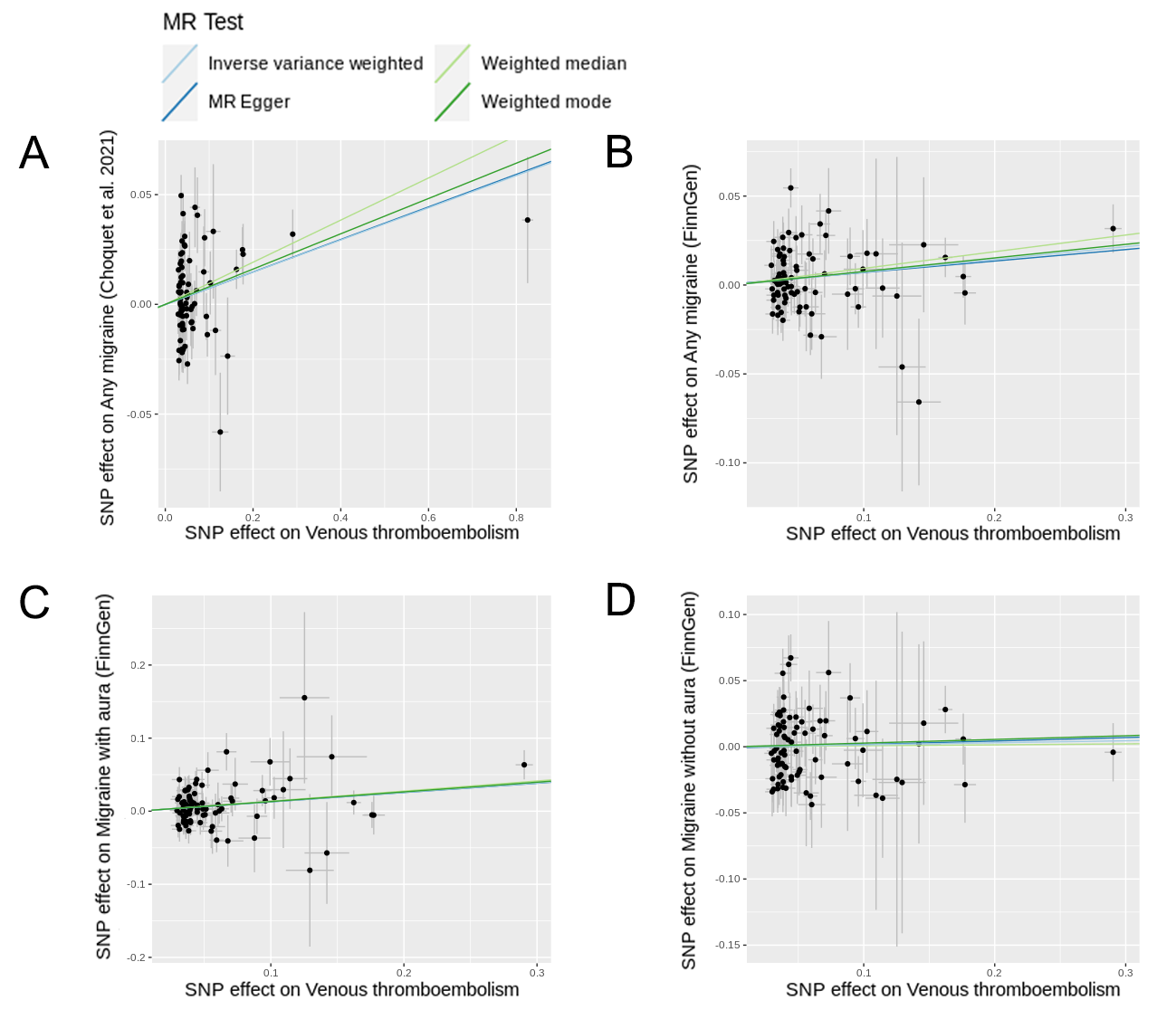


**Supplementary Figure 4 Scatter plots of SNP effects on venous thromboembolism and migraine**

X axes represent SNP effects on migraine. Y axes represent SNP effects on venous thromboembolism.

Abbreviations: MR, Mendelian randomization; SNP, single nucleotide polymorphism


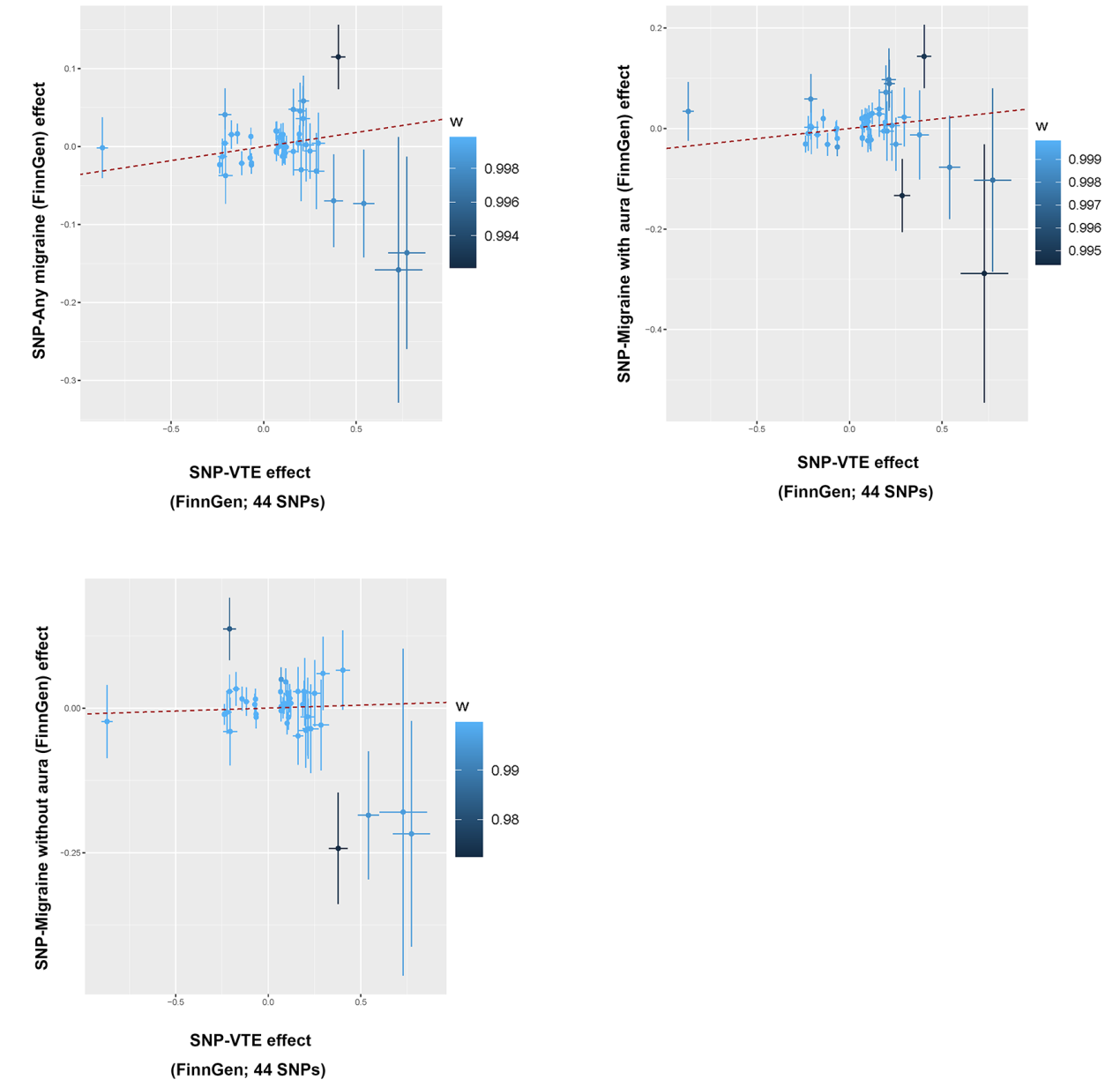


**Supplementary Figure 5 Scatter plots of venous thromboembolism on migraine by exclusively analyzing data from FinnGen**

The Bayesian MR method was used.

All the SNPs utilized exhibited a significant association with VTE at the genome-wide significance level (p-value < 5 × 10^-8^). No outlier was detected by MR-Pleiotropy Residual Sum and Outlier method. All SNPs have a low probability of reverse causality (Steiger p-value < 0.05).

Abbreviations: MR, Mendelian randomization; SNP, single nucleotide polymorphism; VTE, venous thromboembolism.
